# Can pigs add another “P” to the PPR? Serological evidence of frequent Peste des petits ruminants infections in pigs in Nigeria

**DOI:** 10.1186/s13567-025-01482-3

**Published:** 2025-03-05

**Authors:** Adeyinka Jeremy Adedeji, Milovan Milovanovic, Banenat Bajehson Dogonyaro, Jolly Amoche Adole, Mark Samson, David Oludare Omoniwa, Toyin Olubade-Olatokunbo, Logyang Lot Emmauel, Jeremiah Okoro Ijomanta, Kuduk Kakomo Karaye, Elayoni Emmanuel Igomu, Ayokunle Omileye, Helen Onyinyechi Ignatius, Paul Adamu, Valerie Allendorf, Bernd Hoffmann, Clement Meseko, Klaas Dietze

**Affiliations:** 1 National Veterinary Research Institute, Vom, Plateau State Nigeria; 2 Friedrich-Loeffler-Institut, Greifswald, Insel Riems Germany; 3 Department of Veterinary Medicine, Surgery and Radiology, University of Jos, Jos, Plateau State Nigeria; 4 Ministry of Agriculture, Ikeja, Lagos State Nigeria; 5 LHYW Livestock Center, Makurdi, Benue State Nigeria; 6 Ministry of Agriculture, Kafanchan, Kaduna State Nigeria

**Keywords:** Atypical host, disease reservoir, disease eradication, interspecies transmission, PPR, pig

## Abstract

To achieve the global eradication of Peste des petits ruminants (PPR), the epidemiological role of atypical hosts must be fully understood. Among domestic animals, pigs are, until now, the only species that has proven to fulfil criteria relevant for hosts to act as disease reservoir. This entails the susceptibility to infection via contact with infected animals as well as the shedding of infectious virus, resulting in new infections. However, these features have been observed only in infection experiments, lacking information from the field. In this study, for the first time, we provide evidence for frequent PPR virus exposure in pigs, detected in Nigeria. The prevailing husbandry systems targeted for sampling entailed predominantly free roaming pigs and small ruminants. The sampling area was selected on the basis of the occurrence of endemic PPR in small ruminants in recent years. Sera from 183 small ruminants and 495 pigs were analysed. The 25.68% apparent seroprevalence (95% CI 19.5–32.7 at the population level) observed in small ruminants matched values detected in Nigeria. The apparent seroprevalence in pigs of 4.24% (95% CI 2.6–6.5 at the population level) distributed across Nigeria provides evidence that PPR infections in pigs are not rare events. The ability of swine populations to propagate and maintain autonomous PPR infections over time remains to be clarified at this stage. Countries engaged in PPR eradication with substantial pig populations under extensive husbandry practices, including contact with small ruminants, should, however, consider surveillance strategies that address this possibly problematic interspecies interaction.

## Introduction

Peste des petits ruminants (PPR) is currently the only livestock disease addressed by a global eradication program [1], highlighting its paramount importance for livestock sector development. The aim of eradicating PPR by 2030 must be seen as highly ambitious in 2025, with substantial work to be done in regions with endemic PPR occurrence. Its current geographic distribution, which mainly covers large parts of Africa, the near and middle East and central and South Asia [2], matches high small ruminant densities as well as populations engaged in livestock farming in predominantly small-scale settings. Despite the challenging envisaged timeline for PPR eradication, cost‒benefit analyses provide a sound rationale to embark on concerted control and eradication [3]. The well-described socioeconomic impact of PPR on poorer or more vulnerable households [4] provides additional support for such efforts.

Foreseeable difficulties in PPR eradication have been reported by Albina et al. [5]. Many of the highlighted factors remain valid, as they address the complex epidemiology of PPR resulting mainly from diverse hosts as well as some pathogen-determining factors. Its causative agent, PPR virus (PPRV), belongs to the species *Morbillivirus caprinae* and is an RNA virus of the genus *Morbillivirus*. PPRV can be genetically divided into four lineages (I-IV), which cannot be distinguished serologically. Despite being primarily a disease of sheep and goats, PPRV is known to infect a wide range of domesticated and wild animal species.

The long list of wild ruminant species that have been found to be susceptible to PPR [6] raises not only the question of how far the continuous geographic expansion of PPR occurrence has consequences for wildlife conservation efforts [7] but also the extent to which some of these species can act as reservoir hosts. In advanced stages of control and eradication, this potential role has strategic implications for overall success.

Among domesticated animals, camelids and cattle have been studied in experimental settings, and the results indicate that these species can be considered dead-end hosts [8, 9].

The possible role of pigs (*Sus scrofa*) in the epidemiology of PPR has been previously discussed [10]. Earlier experimental work conducted in Nigeria revealed that pigs can undergo subclinical infection after inoculation or contact with infected small ruminants, but the study failed to find evidence for virus transmission from infected pigs to either other pigs or small ruminants [11]. The authors therefore concluded that pigs do not play a role in the epidemiology of PPR, a result challenged four decades later by Schulz et al. [12] revisiting the question of what happens when pigs become inoculated with PPRV or have contact with infected small ruminants. In this more recent study, pigs demonstrated the ability to transmit virus, leading to infection in both pigs and small ruminants despite a relatively short phase of viral shedding and moderate clinical signs. Therefore, pigs and wild boars must be considered as possible reservoir hosts for PPR. This makes further investigations into the role of swine in PPR epidemiology crucial when aiming for global disease eradication.

At the third meeting of the PPR global research and expertise network (GREN), this current state of knowledge was again taken to derive recommendations that call for studies into serological and virological evidence of PPRV infections in atypical hosts [13]. This information is crucial for enabling national control strategies to include generated knowledge in updated surveillance and intervention plans if needed.

Nigeria is one of the countries in West Africa considered endemic for PPR and has reported the circulation of PPRV lineage II and, more recently, predominantly lineage IV [14, 15]. Nigeria hosts the largest small ruminant population in West Africa, with extensive farming systems being the most common, and the occurrence of PPR has always been identified as one key constraint for prosperous small ruminant sector growth [16]. Like small ruminants, the Nigerian pig population is the largest in the region, and despite rapid developments in the pig sector, the vast majority of animals are kept in small-scale husbandry systems with predominantly poor biosecurity measures in place [17, 18].

With the present study, we hypothesized that in husbandry systems where pigs and small ruminants interact frequently through mixed species herding or shared facilities, PPRV circulation in small ruminants will also cross into pig populations, leading to seroconverted pigs of unknown prevalence. To the authors’ knowledge, this is the first time that efforts have been made to gather field data on the epidemiological role of pigs in areas where PPR is considered endemic in small ruminants.

## Materials and methods

The sampling sites were selected through a multistep approach.

1. The mandatory criteria used to fulfil the study purpose were as follows:

- Area where PPR outbreaks were recorded over the past 3 years
- No mass vaccination has been conducted in the past 3 years.
- Prevailing husbandry systems where pigs and small ruminants can have direct contact, such as the sharing of communal livestock grazing and watering points
- Farming communities willing to support the sampling activities

2. The selection of states and their respective local government areas (LGAs) and communities was performed through consultation with state veterinary authorities and the support of the Director of Veterinary Services (DVS).
3. The selection of households and animals was implemented via convenience sampling according to the willingness to contribute by household members and the accessibility of the animals.

Basic data on location, species and age at the time of sampling were recorded from all the animals sampled. The oropharyngeal swabs were collected in viral transport medium (VTM, Vom), and five milliliters of blood were collected in sample tubes (RICHMED 5 mL gel tubes) from pigs and small ruminants. The samples were transported in cool boxes with ice packs from the field to the laboratory in Nigeria, where the cold chain was maintained. For sample transport between laboratories in Nigeria and Germany, a Credo Cube 12 L Series 50 M (Peli Bio Thermal LLC) was used to maintain the cold chain.

For the serological and virological methods, this study used the methods described by Milovanović et al. [19], and the methods description partly reproduces their wording.

### Serological investigations

#### Enzyme-Linked Immunosorbent Assay (ELISA)

A commercially available ID screen PPR competition ELISA kit (ID, Montpelier, France) was used to detect PPRV-specific antibodies from the serum samples according to the manufacturer’s instructions. These instructions indicate different cut-off values to be applied for small ruminants and pigs and have been considered accordingly.

#### Virus neutralization test (VNT)

To assess the titre of the PPRV-specific neutralizing antibodies, a VNT was conducted in 96-well flat-bottomed tissue culture plates in a log_2_ dilution series in triplicate, whereby the positive, negative, and collected serum samples were tested against a constant titre (100 TCID_50_/mL) of the PPRV Nigeria 75/1 vaccine strain. Before titration, all the serum samples were incubated at 56 °C for 30 min for complement inactivation. The serum samples were diluted (from 1:10 to 1:1280) in serum-free cell culture medium (FLI internal medium number: ZB5). The serum dilutions and a fixed amount of the PPRV Niger 75/1 vaccine virus were incubated for 2 h at 37 °C with constant gentle shaking on a tilt shaker. After the neutralization step, a 100 µL suspension of VERO dog SLAM cells (FLI cell culture collection number: RIE1280/57) in ZB5 with 10% fetal calf serum (FCS) and 10 mg/mL Zeozin (InvivoGen, Toulouse, France) was added to each well, and the plates were incubated at 37 °C and 5% CO_2_. Every time the test was performed, one control plate that contained the outcome of the back titration of the test virus and the titration of the positive and negative control sera was generated. After 4 days, the plates were inspected to determine whether the virus cytopathic effect (CPE) was visible, and a final reading was taken on day 7 when the results were recorded. The antibody titre was calculated by using the Spearman and Kaerber method [20]. The samples with a neutralizing titre greater than 10 were considered positive.

### Sample analysis cascade

All the serum samples were tested by ELISA. Samples from pigs that tested positive in the ELISA as well as a subset of negative samples from pigs were retested in the confirmatory VNT. For small ruminants, a subset of positive samples and one negative sample from the ELISA were analysed with the VNT. ELISA were performed in Nigeria, and the subset of samples for the VNT was subsequently analysed in Germany, including a reconfirmation of the ELISA results. All positive samples from the ELISA that were not confirmed in the VNT were counted as negative in further analysis.

### Virological investigations

#### Reverse transcriptase real-time polymerase chain reaction (RT‒qPCR)

For the extraction of viral nucleic acid from sample material, a NucleoMag VET kit (Macherey-Nagel, Düren, Germany) with the half-automated King Fisher platform (King-Fisher Flex magnetic particle processor, Thermo Fisher Scientific, Waltham, MA, USA) was used. For the amplification of the conserved region of the nucleocapsid protein (Np), a specific primer probe was mixed [21] using FAM channel (forward and reverse primer concentrations: 15 µM; probe concentration: 5 µM). As a control for the extraction and amplification process, a heterologous control system [22] was implemented and codetected in all RT‒qPCR runs using the HEX channel (concentrations of forward primer, reverse primer and probe: 5 µM). Commercially available AgPath-ID™ One-Step RT‒PCR Reagents from Applied Biosystems™ (Waltham, MA, USA) were used for real-time RT‒PCR. Briefly, 2.5 µL of template was added to a mixture of 1.25 µL of RNase-free water, 6.25 µL of 2x RT‒PCR Buffer, 0.5 µL of 25x RT‒PCR Enzyme Mix, 1.0 µL of specific NpSRMV primer probe mixture and 1.0 µL of EGFP primer mixture, and RT‒qPCR was run on a CFX96 real‒time PCR cycler (Bio‒Rad, Hercules, CA, USA). Positive and negative controls were used for each run to ensure the validity of the results.

#### Data analysis and statistics

The data were tested for normality using the D'Agostino & Pearson test, and subsequently, the difference in age distribution was assessed using the nonparametric Mann‒Whitney test, with *P* values <0.05 considered significant. The calculations and graphic illustrations were performed with GraphPad Prism version 10.2.1 for Windows (GraphPad Software, Boston, Massachusetts, USA).

Prevalence rates with 95% confidence were calculated according to Clopper-Person using the FLI shiny app [23].

Geospatial data visualization was performed with QGIS Geographic Information System Version 3.34.12 (Open Source Geospatial Foundation).

## Results

**Figure 1:**
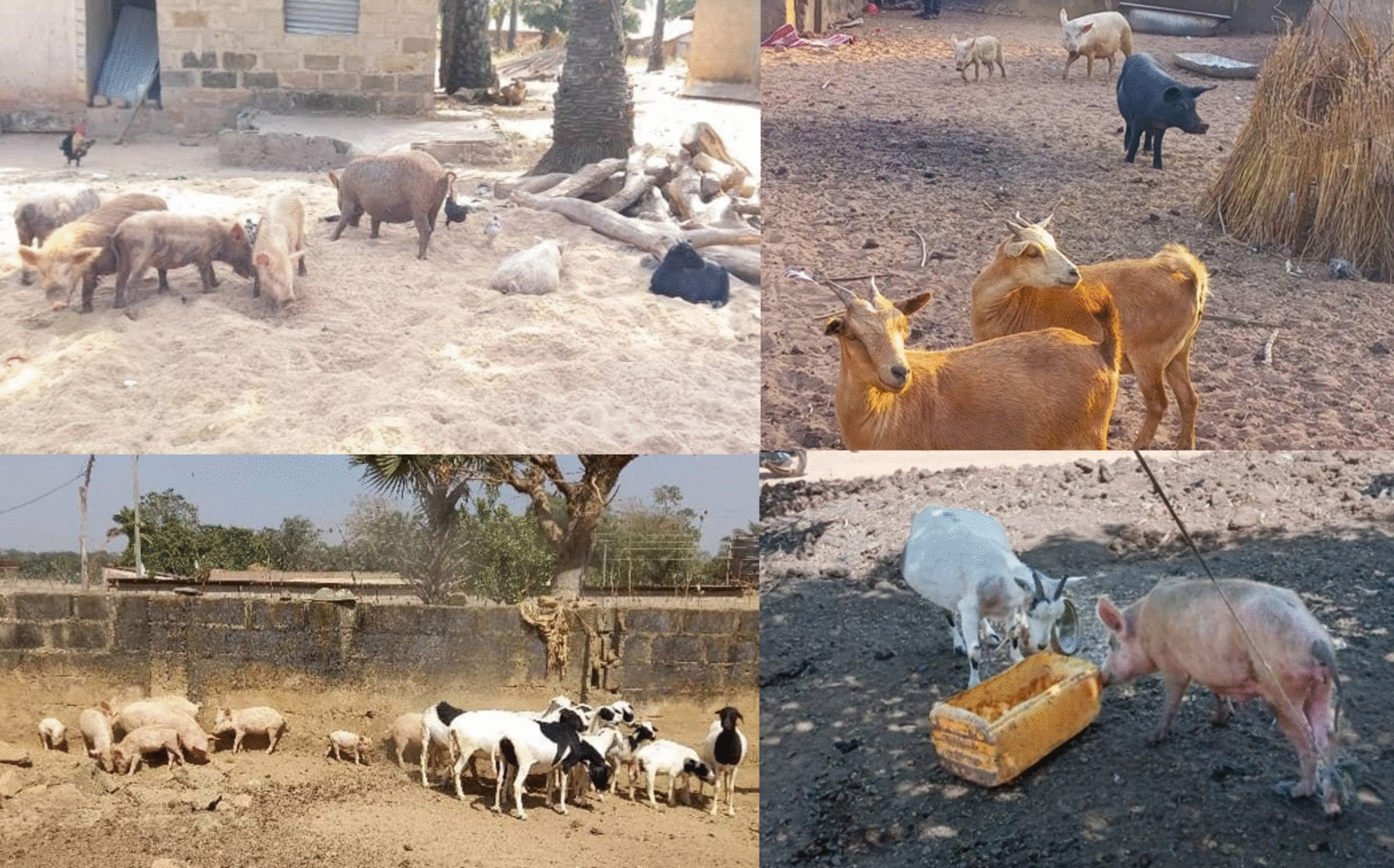
**Prevalent husbandry systems with close interactions between pigs and small ruminants**. Animals in targeted systems often roam together and share feed and water resources.

**Figure 2:**
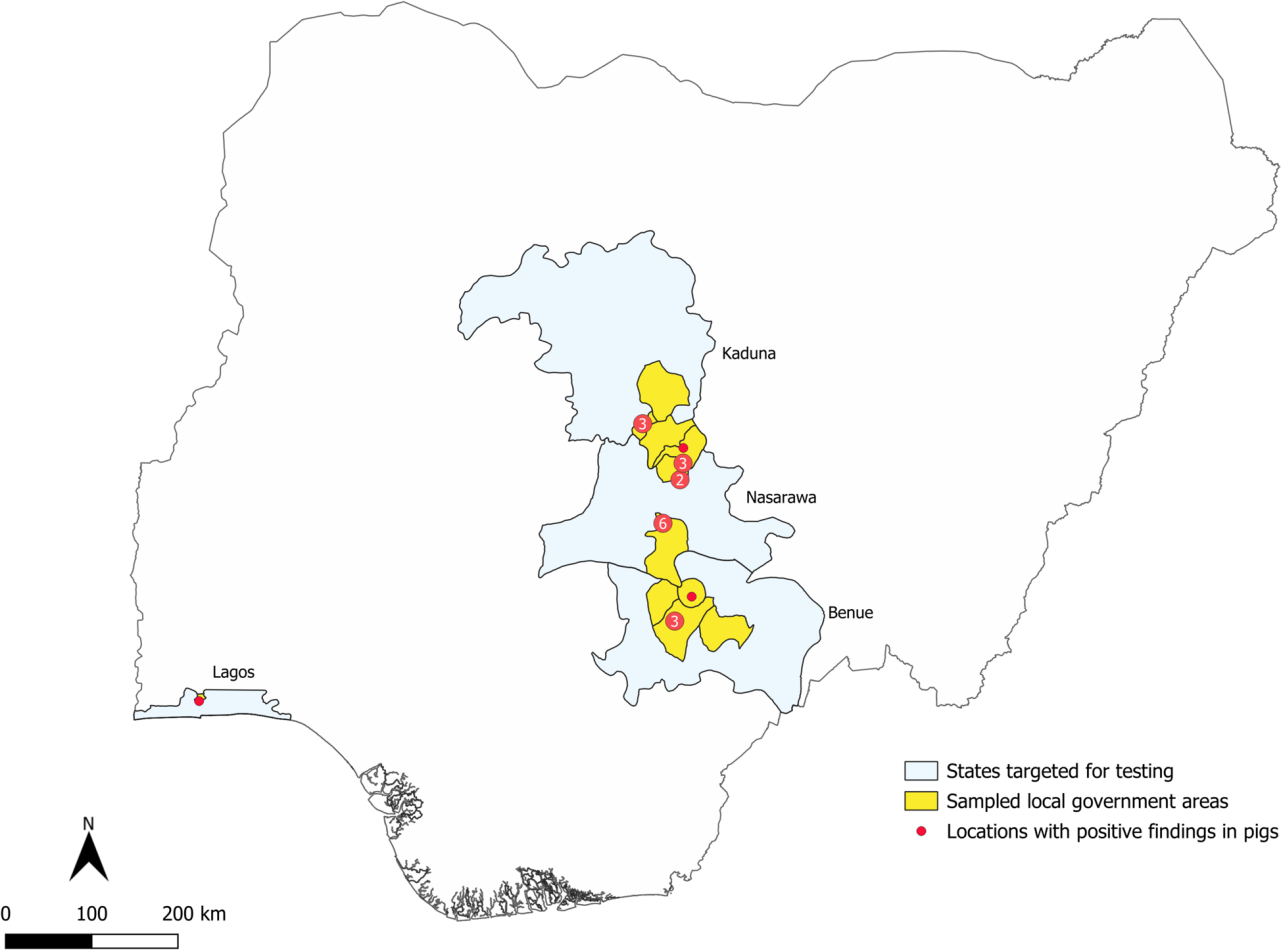
**Map of Nigeria highlighting the states (grey) and local government areas (yellow) where sampling was conducted.** Locations of positive findings in pigs are marked by red dots, and the numbers on the dots indicate the underlaying number of locations if more than one.

Samples were collected between December 2022 and January 2023, which is the dry season in Nigeria and the period when livestock were released to roam freely in rural agrarian communities. During the sampling period, 183 small ruminants and 495 pigs were sampled. The animals were distributed across the four federal states Benue, Kaduna, Nasarawa, and Lagos. Examples of the target husbandry systems are shown in Figure 1. The geographic expansion of the sampling activity and the locations of positive findings in pigs are depicted in Figure 2. To define the flocks for sampling in the target communities, farmers were asked whether their flock had had suspected or confirmed outbreaks of PPR in the past 6–12 months and whether no vaccination had been performed. Only flocks for which both criteria met the sampling criteria were included.

Overall serological findings and calculated seroprevalence rates are summarized in Table 1.

Table 1. For small ruminants, the detected apparent seroprevalence was 25.68%, whereas for pigs, this value was 4.24%.

**Table 1:** **Summary of serological findings and calculated prevalence (Clopper-Pearson, 95% confidence)**. For pigs, only VNT confirmed positive samples were counted as positive.

	Individuals sampled	negative	positive	Prevalence (%)	CI (95%)
Small ruminants	183	136	47	25.68	19.5–32.7
Pigs	495	474	21	4.24	2.6–6.5

**Figure 3:**
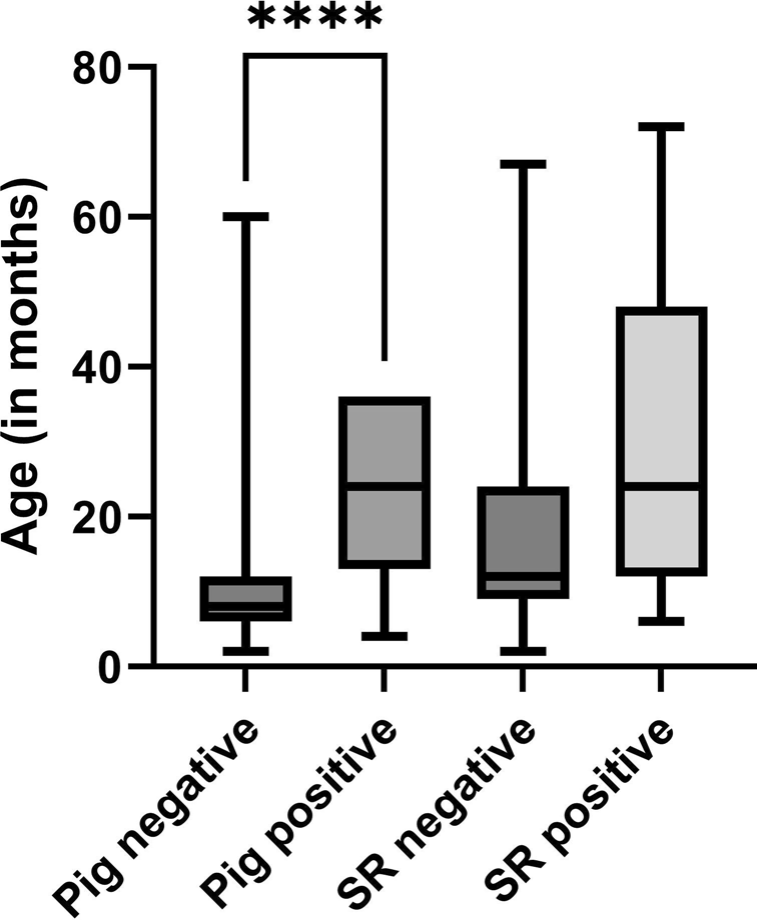
**Age distribution of the sampled animals grouped by species (SR= small ruminant) and serological results**. ****p value < 0.0001. For pigs, only VNT-confirmed positive samples were considered positive.

An overview of the age distributions of the sampled animals divided by species and serological results is given in Figure 3.

**Figure 4:**
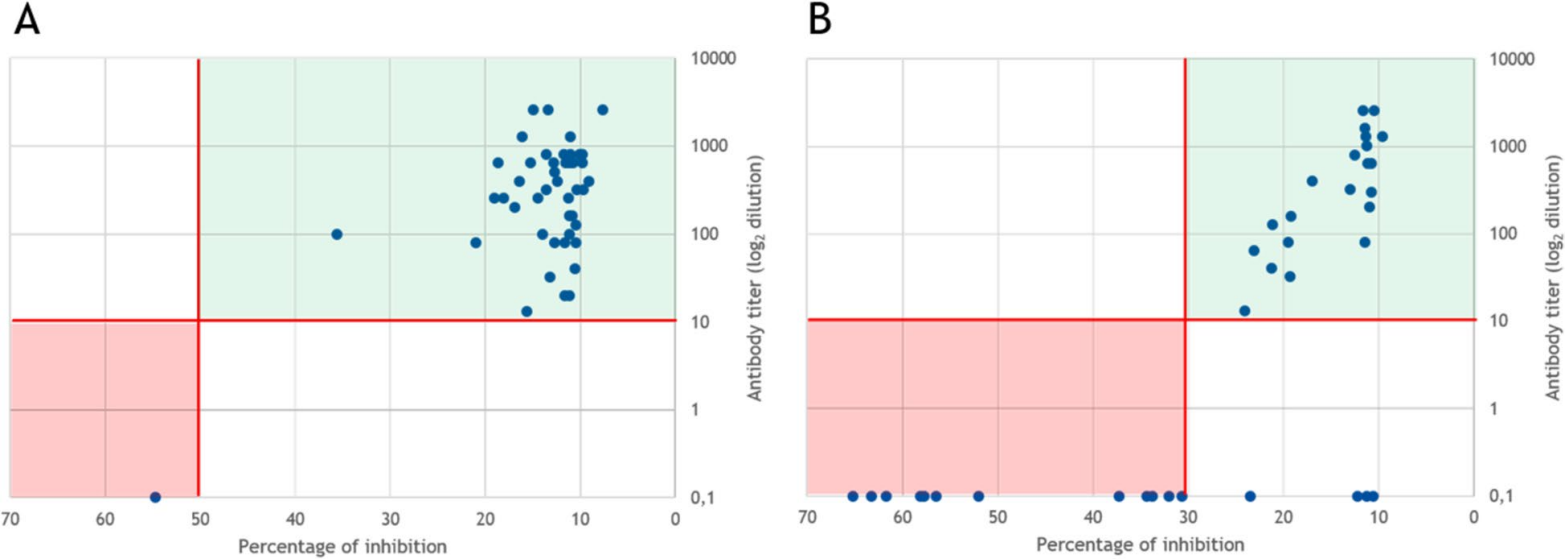
**Comparison of serological methods (ELISA and VNT) for subsets of samples with readout values**. X-axis: percentage of inhibition, cut-off value provided by the manufacturer (50% in small ruminants and 30% in pigs); Y-axis: antibody titre (log_2_ dilution), cut-off value of 10. The shaded area indicates congruent results in both tests: green positive and red negative. **A** Results for small ruminant samples and **B** for pig samples.

A comparison of the readout values of the ELISA-based antibody detection and the corresponding VNT titres is provided in Figure 4. Four of the 26 porcine samples that tested positive by ELISA with inhibition percentages well apart from the cut-off value could not be confirmed by the VNT. In this study, samples not confirmed in the VNT were considered negative. Porcine serum samples that tested positive had antibody titres comparable to the levels found in small ruminants.

The detection of viral genome fragments was not possible in any of the obtained swab samples from pigs, and only one swab sample from one small ruminant had a positive result (ct value of 30.35).

## Discussion

The detected apparent PPR seroprevalence in small ruminants of 25.68% falls within the expected range for Nigeria, as reviewed by Esonu et al. [24]. Our sample set therefore reflects very well the endemic PPR setting in small ruminants encountered in the selected states. The apparent seroprevalence of 4.24% in pigs is comparable to the reported prevalence for camels as atypical host in northern Nigeria [25]. Pigs were found to be positive in all four states included in this study, covering a wider geographic range. This finding points towards widespread interspecies interactions and virus spillover in the selected favourable scenario. More targeted surveillance approaches could provide indications of whether pigs are able to propagate the infection after the spill over in those respective settings.

The commercial kit used for antibody detection has been shown to perform well in small ruminant samples. This can be seen as reconfirmed when the test results are compared with the subset of samples that were tested by VNT. For pig samples, although the manufacturer provides adapted cut-off values, comparison with the VNT as a confirmatory test revealed reduced specificity. However, whether cross-reactivity with antibodies against other known or unknown morbilliviruses plays a role remains to be determined. For example, a more recently described porcine morbillivirus [26] has thus far been detected in only Mexico; however, more epidemiological studies are needed to determine the overall distribution patterns of this virus.

The tendency of seropositive animals to be older was expected, confirming the cumulative nature of seropositivity at the population level over time, as antibodies are detectable after natural infection or vaccination for several years [27, 28]. These results indicate that this epidemiological observation applies to pigs alike.

As the sampling approach was primarily designed to identify seroconverted animals, the low level of detection of the viral genome is not surprising. The targeted species, sheep and goats, are perceived to clear the infection with PPRV with no described carrier stages [29]. For pigs, such data do not exist, but the obtained results indicate a comparable setting.

Recent work from Rahman et al. [30], which analysed PPRV genome sequences obtained from wild species and atypical hosts and compared them with PPRV genome sequences obtained from the traditional host species sheep and goats, revealed that the sequences are closely related within the same geographic area and that no distinct clustering in relation to the species involved was observed. None of the atypical host species included had been confirmed as reservoir hosts. As no PPRV sequences obtained from pigs exist in public databases, these findings need to be verified in pigs, but a similar result could be anticipated.

The described husbandry systems for pigs and small ruminants in West Africa have been shown to be a realistic setting for pathogens to be transmitted between these different species. Considering the substantial growth in livestock production observed, e.g., in Nigeria, with an over ninefold increase in pigs between 1982 and 2022 matched with an approximately sixfold increase in small ruminants in the same period [31], this interface has significantly gained importance and will need to be considered in disease control programs generally. As mixed-species live animal markets remain a dominant component in the livestock value chains of the country, with known consequences for disease spread in both small ruminants and pigs [32, 33], efforts to understand possible PPR dynamics between pigs and small ruminants will need to extend beyond the local farming setting.

This study provides the first serological field evidence of PPRV exposure in pigs. In a setting where small ruminants and pigs are in close contact, this must be considered an important finding when aiming for global PPR eradication. This finding calls for an update of the formulation on PPR host species of the WOAH [29] stating “Pigs have been reported as being susceptible and transmitting the virus under laboratory conditions but thus far not in the field”. The epidemiological relevance of the detected exposure remains to be clarified. Countries in the process of PPR control with prevailing production systems where pigs and small ruminants interact are urged to consider pigs as potential reservoir species in the advanced stages of eradication, unless further investigations reveal pigs as epidemiological dead-end hosts under field conditions. In particular, in the latter case, pigs could serve as sentinels in later stages of eradication programs, as is discussed for cattle [34], despite the comparably lower detected seroprevalence rates, as they would remain susceptible, unvaccinated populations.

## Data Availability

The datasets used and/or analysed during the current study are available from the corresponding author upon reasonable request.
